# Multispecies probiotic supplementation in diet with reduced crude protein levels altered the composition and function of gut microbiome and restored microbiome-derived metabolites in growing pigs

**DOI:** 10.3389/fmicb.2023.1192249

**Published:** 2023-07-07

**Authors:** Robie Vasquez, Sang Hoon Kim, Ju Kyoung Oh, Ji Hoon Song, In-Chan Hwang, In Ho Kim, Dae-Kyung Kang

**Affiliations:** Department of Animal Biotechnology, Dankook University, Cheonan, Republic of Korea

**Keywords:** probiotics, crude protein, gut microbiome, short-chain fatty acid, polyamine, pig

## Abstract

Both crude protein (CP) and probiotics can modulate the gut microbiome of the host, thus conferring beneficial effects. However, the benefits of low CP diet supplemented with multispecies probiotics on gut microbiome and its metabolites have not been investigated in pigs. Thus, we investigated the combinatory effects of low CP diet supplemented with multispecies probiotics on gut microbiome composition, function, and microbial metabolites in growing pigs. In total, 140 6 week-old piglets (Landrace × Yorkshire × Duroc) were used in this study. The pigs were divided into four groups with a 2 × 2 factorial design based on their diets: normal-level protein diet (16% CP; NP), low-level protein diet (14% CP; LP), NP with multispecies probiotics (NP-P), and LP with multispecies probiotics (LP-P). After the feeding trial, the fecal samples of the pigs were analyzed. The fecal scores were improved by the probiotic supplementation, especially in LP-P group. We also observed a probiotic-mediated alteration in the gut microbiome of pigs. In addition, LP-P group showed higher species richness and diversity compared with other groups. The addition of multispecies probiotics in low CP diet also enhanced gut microbiota metabolites production, such as short-chain fatty acids (SCFAs) and polyamines. Correlation analysis revealed that Oscillospiraceae UCG-002, *Eubacterium coprostanoligenes*, Lachnospiraceae NK4A136 group, and Muribaculaceae were positively associated with SCFAs; and *Prevotella, Eubacterium ruminantium, Catenibacterium, Alloprevotella,* Prevotellaceae NK3B31 group, *Roseburia, Butyrivibrio,* and *Dialister* were positively correlated with polyamines. Supplementation with multispecies probiotics modulated the function of the gut microbiome by upregulating the pathways for protein digestion and utilization, potentially contributing to enriched metabolite production in the gut. The results of this study demonstrate that supplementation with multispecies probiotics may complement the beneficial effects of low CP levels in pig feed. These findings may help formulate sustainable feeding strategies for swine production.

## 1. Introduction

Diet has a substantial impact on the health and performance of livestock (Tilocca et al., 2017). At livestock farms, diet composition is continually optimized to achieve maximum yield in terms of animal product quality and profitability. A balance between the proportions of carbohydrates (fiber) and crude protein (CP) in feed, along with sufficient proportions of essential vitamins and minerals, is vital to improve livestock performance. Numerous studies have demonstrated the impact of dietary CP content on animals, especially pigs (Chen et al., 2018; Yu et al., 2019; Liu et al., 2022). The metabolic products of protein fermentation play important regulatory roles in maintaining the health of the host (Rist et al., 2013; Pieper et al., 2016). However, excess dietary CP concentration has negative implications not only for animal growth and health but also for the environment. Previous studies have reported that reduction of CP content in diet improves post-weaning diarrhea in pigs (Nyachoti et al., 2006; Heo et al., 2010; Lynegaard et al., 2021), as well as improve swine intestinal health (Fan et al., 2017; Chen et al., 2018). In addition, lower dietary CP reduces the excretion of malodorous compounds to the environment (Clark et al., 2005; Cho et al., 2015). A low protein diet also improves meat quality (Zhu et al., 2022). Recent studies have also shown that reduction in dietary CP leads to modulation of the gut microbiome in pigs, as well as its metabolites (Fan et al., 2017; Chen et al., 2018; Yu et al., 2019; Zhu et al., 2022). This feeding strategy can be advantageous but can also negatively impact the growth performance of pigs. Several studies have revealed that CP reduction impaired the growth performance of pigs potentially due to inhibition of digestion enzymes activity and retarded villus morphology (Yu et al., 2019).

Thus, low CP diet is usually coupled with other feeding strategies such as supplementation of digestible amino acids (Opapeju et al., 2008; Millet et al., 2018), antibiotics (Zhang et al., 2016), enzymes (such as phytase and xylanase) (Atakora et al., 2011), or probiotics (Bhandari et al., 2010; Tang et al., 2019). The supplementation of livestock feed with probiotics is becoming popular due to its positive impact on production yield and livestock health (Barba-Vidal et al., 2019). Probiotics exert beneficial effects on animals, such as improving growth performance, feed efficiency, meat quality, and alleviating weaning stress and diarrhea (Zimmermann et al., 2016; Barba-Vidal et al., 2019). Multispecies probiotics, on the other hand, are a combination of microbes “containing strains of different probiotic species that belong to one or preferentially more genera” (Timmerman et al., 2004). Ideally, each component of this type of probiotic complements each other, increasing its effectiveness as a probiotic. Previous studies have reported that the supplementation of multispecies probiotics in pig feed is beneficial for the overall health and performance of pigs (Timmerman et al., 2004; Mori et al., 2011; Kwak et al., 2021).

The gut microbiome has been extensively studied to develop strategies for improving livestock production quality and yield, and for reducing the excretion of malodorous compounds (Nowland et al., 2019). Changes in the gut microbiome and its metabolites have either beneficial or deleterious effects on the health of growing pigs, thus affecting their growth performance (Davila et al., 2013; Luise et al., 2021). The gut microbiome-derived metabolites, such as short-chain fatty acids (SCFAs), branched chain fatty acids (BCFAs), and polyamines, are highly influenced by CP levels and supplementation of probiotics in the diet (Davila et al., 2013; Pieper et al., 2016; Luise et al., 2021; Vasquez et al., 2022). In our previous study, we explored the effects of multispecies probiotics on the gut microbiome of growing pigs (Oh et al., 2021). However, insufficient data are available regarding the effects of low CP diet supplemented with multispecies probiotics on the gut microbiome, as well as on the production of metabolites, such as SCFAs and polyamines, by the pig gut microbiome. Therefore, in the present study, we investigated the potential benefits of supplementing multispecies probiotics to low CP diet on the gut microbiome and microbial metabolites of growing pigs.

## 2. Materials and methods

### 2.1. Experimental design, animals, and diets

All animal protocols used in this study were approved by the Dankook University Animal Care Committee (Approval number: DK-2-2018). A total of 140 piglets (Landrace × Yorkshire × Duroc) weaned at 4 weeks of age were used in this study. The average body weight of the piglets was 25.01 ± 1.79 kg. All animals were fed the same basal diet after weaning for 2 weeks. The composition of the basal diet (Table 1) was formulated to meet or exceed the National Research Council (NRC)-recommended nutrition for pigs weighing 25–50 kg (National Research Council, 2012). The pigs were randomly divided into the following four groups with a 2 × 2 factorial design based on their diet: normal-level protein diet (16% CP, NP), low-level protein diet (14% CP, LP), NP with multispecies probiotic supplementation (NP-P), and LP with multispecies probiotic supplementation (LP-P). The multispecies probiotic was composed of *Bacillus amyloliquefaciens* G10 (2.5 × 10^8^ colony forming units (CFU)/g feed), *Levilactobacillus brevis* M10 (1.2 × 10^8^ CFU/g feed), *Bacillus subtilis* (3.3 × 10^8^ CFU/g feed), and *Limosilactobacillus reuteri* RTR (1.2 × 10^8^ CFU/g feed). These species have been previously selected for their potential probiotic characteristics (Oh et al., 2021). All pigs were housed in an environmentally controlled room. Each pen contained 4–5 pigs and was equipped with a one-sided self-feeder and a nipple waterer for *ad libitum* access to feed and water. The feeding period lasted for 6 weeks. Fecal score was evaluated daily according to the following criteria: (1) hard and dry pellets but low mass, (2) hard and formed stool, (3) soft and formed stool but moist, (4) soft and unformed stool, and (5) watery, liquid stool (Park et al., 2018). On the last day of the experiment, fresh fecal samples were individually collected from the rectum of the pigs in each group and stored at −80°C for further analyses. For pH analysis, the fecal samples (0.5 g) were homogenized in distilled water, supernatant was collected after centrifugation at 10,000 × *g* for 10 min, and the pH was determined using a pH meter (Horiba, Japan). The moisture content of the fecal samples (0.5 g) was measured using a moisture analyzer (Kett, Japan).

**Table 1:** Ingredients and chemical composition of the basal pig feed (as-fed basis).

Composition	Experimental diets	
	NP	LP
Ingredients (%)		
Corn	74.99	79.87
Soybean meal (48%)	20.0	14.6
Tallow	1.88	1.99
DCP	1.28	1.38
Limestone	0.73	0.71
Salt	0.20	0.20
Methionine (99%)	0.08	0.10
Lysine	0.46	0.65
Threonine (99%)	0.13	0.22
Tryptophan (99%)	0.02	0.05
Mineral mix^a^	0.10	0.10
Vitamin mix^b^	0.10	0.10
Choline	0.03	0.03
Total	100	100
Calculated values		
Crude protein (CP), %	16.0	14.0
Ca, %	0.66	0.66
P, %	0.56	0.56
Lys, %	1.12	1.12
Met, %	0.32	0.32
Thr, %	0.72	0.72
Trp, %	0.19	0.19
ME, kcal/kg	3,300	3,300
Fat, %	4.75	4.95
Fiber, %	2.48	2.38
Ash, %	4.48	4.29

NP, normal-level protein diet; LP, low-level protein diet.

a Provided per kg of complete diet: Fe, 138 mg as ferrous sulfate; Cu, 84 mg as copper sulfate; Mn, 24 mg as manganese oxide; Zn, 72 mg as zinc oxide; I, 0.6 mg as potassium iodide; and Se, 0.36 mg as sodium selenite.

b Provided per kg of complete diet: vitamin A, 15,600 IU; vitamin D3, 2,040 IU; vitamin E, 72 IU; vitamin K3 6 mg; thiamine, 4 mg; riboflavin, 20 mg; pyridoxine, 6 mg; vitamin B12, 8.04 mg; niacin, 0.66 mg; Ca-pantothenate, 54 mg; folic acid, 2.52 mg; and biotin, 0.40 mg.

### 2.2. Preparation of the multispecies probiotic

The multispecies probiotic was prepared as described by Oh et al. (2021). Briefly, a solid-state fermentation process was used to prepare the probiotic. Subsequently, the probiotic was processed into powdered form and mixed with the basal feed to achieve the final individual doses. Genebiotech (Seoul, South Korea) performed the preparation of the multispecies probiotics. Feed mixed with the probiotic was kept in a sterile container at 4°C. The viability of the probiotic was assessed daily.

### 2.3. Determination of fecal metabolite levels (SCFAs, BCFAs, lactate, and polyamines)

We performed high performance liquid chromatography (HPLC) to determine the levels of lactate, SCFAs (acetate, propionate, butyrate, and valerate), and BCFAs (isobutyrate and isovalerate) in the fecal samples; the samples for HPLC were prepared as previously described (Zhang et al., 2022). Briefly, the fecal samples (0.5 g) were suspended in 1 mL of sterile demineralized water, vortexed for 3 min, and centrifuged at 10,000 × *g* for 10 min at 4°C. The supernatant was collected and filtered using 0.22-μm PTFE syringe filters. The samples were analyzed using an Agilent Infinity 1260 HPLC System (Agilent, United States) with Aminex HPX-87H column (300 × 7.8 mm; Bio-Rad, United States), equipped with refractive index and UV detectors (*λ* = 210 nm). The samples (10 μL) were injected using an autosampler, while the column temperature was maintained at 65°C. The mobile phase was 0.005 M H_2_SO_4_, and the flow rate was maintained at 0.6 mL/min for a total run time of 35 min.

The fecal samples for the analysis of polyamines (putrescine, cadaverine, histamine, spermidine, and spermine) were prepared according to previously described derivatization methods (Yoon et al., 2015; Li et al., 2018), with slight modifications. Briefly, the fecal samples (0.5 g) were suspended in 1.5 mL of 0.4 M perchloric acid (Sigma-Aldrich) and vortexed thoroughly, the suspension was centrifuged at 13,000 × *g* for 10 min, and the supernatant was collected. Next, 2 M NaOH and saturated NaHCO_3_ were added to the supernatant, the solution was reacted with dansyl chloride (5 mg/mL in acetone; Daejung, South Korea) at 50°C for 45 min. The reaction was stopped by adding 25% NH_4_OH and incubation at 50°C for 15 min. The volume of the reaction mixture was adjusted to 1.5 mL using acetonitrile (JT Baker, PA, United States), followed by centrifugation at 2,500 × *g* for 5 min. The supernatant was collected and filtered using 0.22-μm PTFE syringe filters. Samples (10 μL each) were analyzed using an Agilent Infinity 1260 HPLC System (Agilent, United States) equipped with a C-18 reversed phase (150 × 4.6 mm, 5 μm) column (Young Jin Biochrom, South Korea) and a UV detector (*λ* = 254 nm). Acetonitrile and water were used as the mobile phases (linear gradient). The column temperature was set at 30°C, and the flow rate was maintained at 0.8 mL/min for a total run time of 35 min. The concentration of each metabolite was evaluated against a calibration curve generated using standards. All standards were purchased from Sigma-Aldrich (St. Louis, MO, United States).

### 2.4. 16S rRNA gene sequencing and microbial community analysis

Genomic DNA was extracted from fecal samples using the QiaAmp PowerFecal Pro DNA Kit (Qiagen, Hilden, Germany) according to the manufacturer’s instructions. The concentration and purity of the genomic DNA were determined using a UV spectrophotometer (Molecular Devices, CA, United States). Illumina MiSeq (Illumina, CA, United States) platform was used to amplify the V3–V4 hypervariable region of the 16S rRNA gene (CJ BioScience, Inc., Seoul, South Korea). The obtained raw sequencing data were processed using the Quantitative Insights Into Microbial Ecology (QIIME2) pipeline (Bolyen et al., 2019). Primers and adapters were removed from the raw sequences using the “cutadapt” plugin in QIIME2 (Martin, 2011). Sequence quality control and feature table construction were performed using DADA2 (Callahan et al., 2016). Phylogenetic diversity analyses were performed using the “q2-phylogeny” and “q2-diversity” plugins’; the feature classifiers were trained using the “q2-feature-classifier” plugin in within QIIME2, using the SILVA 138_99 database (Quast et al., 2013). Principal coordinate analysis (PCoA) based on Bray–Curtis distance matrix was performed using the “q2-diversity” plugin in QIIME2. Alpha diversity indices (Chao1, Shannon entropy, and Simpson indices), PCoA plot, and relative abundance bar graphs were constructed using the “ggplot2” package in R program v.4.0.2 (Core R Team, 2019). All metagenomic data generated in this study have been deposited at the National Center for Biotechnology Information (NCBI) Sequence Read Archive (SRA) (BioProject accession number: PRJNA856074).

Differential taxonomic markers for each group were determined using the “run_lefse” package (Cao, 2020) in R program based on Linear discriminant analysis effect size (LEfSe) (Segata et al., 2011). Functional prediction based on the Kyoto Encyclopedia of Genes and Genomes (KEGG) database was performed using Phylogenetic Investigation of Communities by Reconstruction of Unobserved States (PICRUSt2) (Douglas et al., 2020). Correlation analyses were performed by calculating Pearson correlation coefficient using the “Hmisc” package in R, and the data were visualized using the “pheatmap” package in R (Core R Team, 2019).

### 2.5. Statistical analyses

Statistical analyses of microbial composition and metabolite concentration data were performed using R (Core R Team, 2019). The normality of data distribution was analyzed using the Shapiro–Wilk test. Multivariate analysis of variance (MANOVA) was used to calculate the effects of CP levels, probiotics, and their interactions. One-way analysis of variance (ANOVA) with Tukey’s test was used to analyze significant differences among treatments. False discovery rate (FDR) correction was performed as necessary. The Kruskal–Wallis test for alpha and beta diversity was performed using the QIIME2 pipeline. Permutational multivariate analysis of variance (PERMANOVA) was used to determine significant differences in the PCoA plot. Data were considered significant at *p* < 0.05.

## 3. Results

### 3.1. Effect of multispecies probiotic on pH and moisture content of fecal samples

Fecal sample analyses (Table 2) revealed that pH significantly decreased with probiotic supplementation (*p* < 0.001). Similarly, the moisture content and fecal scores were significantly improved after probiotic supplementation, irrespective of the CP level (*p* = 0.04 and *p* < 0.01, respectively). Moreover, LP-P group exhibited significantly lower moisture content and better fecal scores than the LP group (*p* < 0.05).

**Table 2:** Effect of dietary probiotic supplementation on fecal scores and pH moisture content of fecal samples of growing pigs.

Items	Treatments^1^				SEM	*p* values^2^		
	NP	LP	NP-P	LP-P		CP	Pro	CP × Pro
pH	6.55	6.50	6.39	6.33	0.03	0.27	<0.001	0.90
Moisture content, %	23.20	24.36^a^	23.28	22.89^b^	1.76	0.18	0.02	0.007
Fecal score	2.63	2.91^a^	2.57	2.40^b^	0.65	0.59	0.008	0.03

Significant differences among treatments were determined using Kruskal-Wallis test. Mean values within a row without a common superscript differ significantly (*p* < 0.05). NP, normal-level protein diet; LP, low-level protein diet; NP-P, normal-level protein diet + probiotic; LP-P, low-level protein diet + probiotic; SEM, standard error of mean; CP, crude protein; Pro, probiotics.

1 Values were reported as mean (*n* = 35 each treatment).

2 *p* values were calculated using multivariate ANOVA (MANOVA).

### 3.2. Effect of multispecies probiotic on fecal metabolites

To investigate the effect of multispecies probiotic supplementation in feed with normal or low CP level on gut microbiome-derived metabolites, we measured the lactate, SCFA, BCFA, and polyamine levels in the fecal samples of growing pigs (Table 3). The acetate, propionate, and valerate concentrations of the LP group were lower than those of the NP group. Probiotic supplementation, regardless of CP level, significantly increased fecal acetate, propionate, and valerate levels (*p* = 0.01, *p* = 0.04, and *p* = 0.006, respectively). Interestingly, fecal SCFA levels of LP-P were comparable with NP-P compared with LP alone. However, significant changes were not observed in fecal butyrate and lactate levels, and no differences were observed in fecal isobutyrate and isovalerate levels. The polyamine (putrescine, cadaverine, spermidine, and spermine) levels of the LP group were reduced compared to that in the other groups; however, the differences were not significant (*p* > 0.05). By comparison, NP-P and LP-P marginally increased the concentrations of these polyamines (*p* > 0.05).

**Table 3:** Effect of dietary probiotic supplementation on microbiome-derived metabolites.

Items	Treatments^1^				SEM	*p* values^2^		
	NP	LP	NP-P	LP-P		CP	Pro	CP × Pro
SCFA, μmol/g								
Acetate	321.73^a^	342.08	372.58	415.00^b^	13.09	0.15	0.01	0.77
Propionate	17.48	16.50^a^	19.81	22.20^b^	0.75	0.06	0.04	0.91
Butyrate	14.37	12.78	13.48	13.44	0.55	0.89	0.85	0.54
Valerate	2.63	1.90^a^	3.50	4.24^b^	0.39	0.04	0.006	0.19
Lactate, μmol/g	167.32	209.40	174.27	205.82	10.18	0.18	0.40	0.72
BCFA, μmol/g								
Isobutyrate	1.14	0.91	1.37	1.94	0.23	0.52	0.37	0.38
Isovalerate	4.10	4.26	4.87	4.84	0.30	0.16	0.95	0.91
Polyamines, μg/g								
Putrescine	55.50	48.87	55.45	70.13	4.19	0.42	0.77	0.13
Cadaverine	54.39	36.19	52.80	58.36	4.10	0.77	0.81	0.04
Histamine	2.48	2.62	3.14	3.27	0.17	0.39	0.05	0.33
Spermidine	130.05	107.17	130.78	144.11	7.20	0.91	0.90	0.23
Spermine	6.97	6.69	6.81	7.86	0.23	0.08	0.73	0.27

Significant differences among treatments were determined using Kruskal-Wallis test. Mean values within a row without a common superscript differ significantly (*p* < 0.05). NP, normal-level protein diet; LP, low-level protein diet; NP-P, normal-level protein diet + probiotic; LP-P, low-level protein diet + probiotic; SEM, standard error of mean; CP, crude protein; Pro, probiotics.

1 Values were reported as mean (*n* = 35 each treatment).

2 *p* values were calculated using multivariate ANOVA (MANOVA).

### 3.3. Effect of probiotics on gut microbial structure and composition

**Figure 1:**
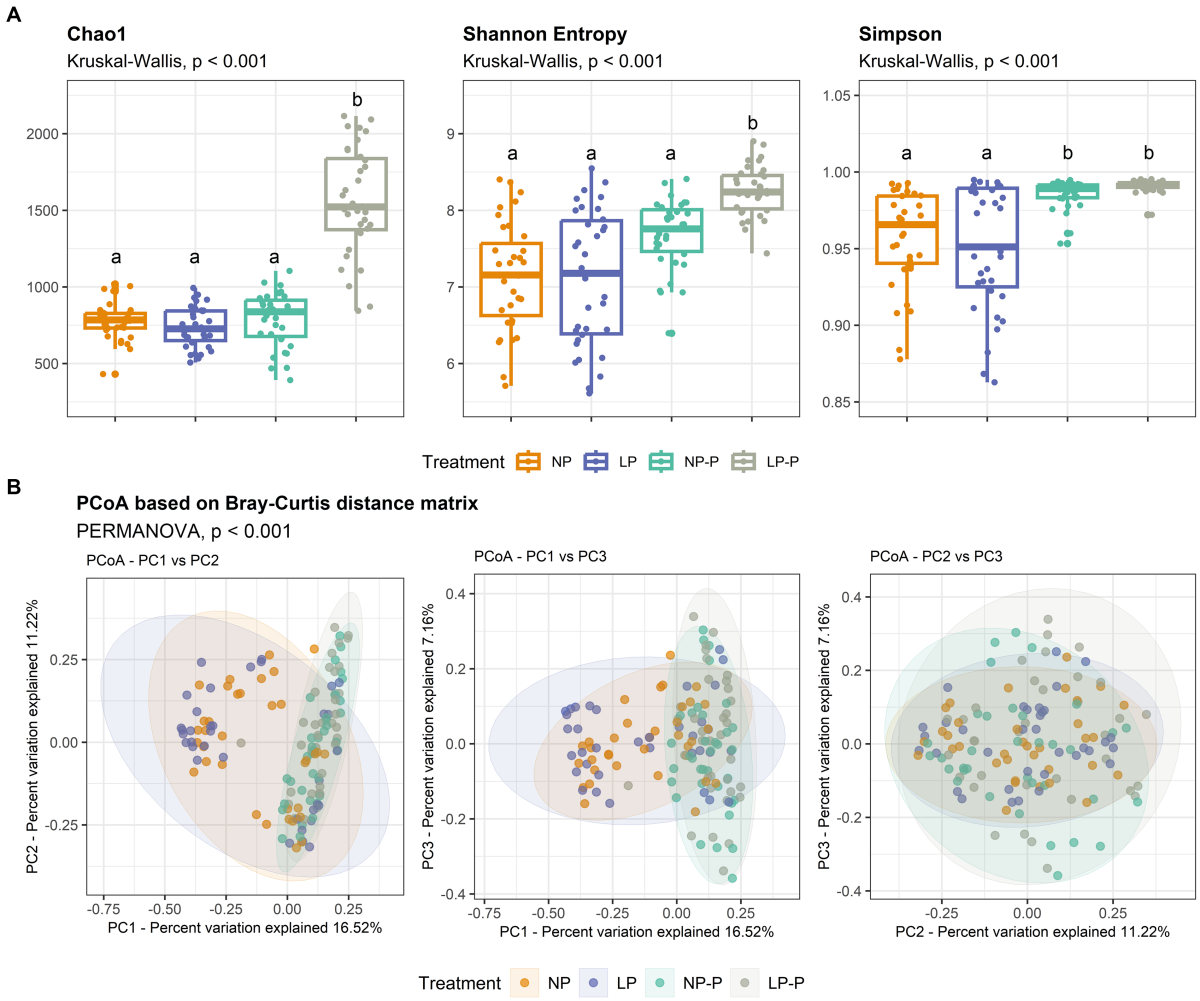
Changes in the gut microbiome structure. Comparison of alpha-diversity indices Chao1, Shannon entropy, and Simpson **(A)**. Significant differences among treatments were determined using Kruskal-Wallis test. Principal coordinate analysis (PCoA) plot based on Bray-Curtis distance matrix **(B)**. NP, normal-level protein diet; LP, low-level protein diet; NP-P, normal-level protein diet + probiotic; LP-P, low-level protein diet + probiotic.

The gut microbiome structure and community composition of the growing pigs from all four groups were examined. Sequencing of the 16S rRNA yielded 32,171,215 raw reads for 140 samples, which contained 15,415,965 valid reads after filtering and removing chimeric reads. The average length of valid reads was 412 bp. A rarefaction curve was generated to check the appropriateness of the reads for downstream analyses (Supplementary Figure S1). Alpha diversity analyses (Figure 1A; Supplementary Table S1) revealed that the LP-P group showed significantly higher species richness than the other treatment groups, as revealed by the Chao1 index (*p* < 0.001). Similarly, the species diversity in the LP-P group was significantly higher than that in the other treatment groups, based on the Shannon index (*p* < 0.001); and both the NP-P and LP-P groups exhibited increased species evenness compared to that of the LP and NP groups, as revealed by the Simpson index (*p* < 0.001). On the other hand, PCoA based on the Bray–Curtis distance matrix (Figure 1B) revealed distinct clusters between diets with and without probiotics (*p* < 0.001). No significant differences were observed between NP and LP, or NP-P and LP-P groups.

**Figure 2:**
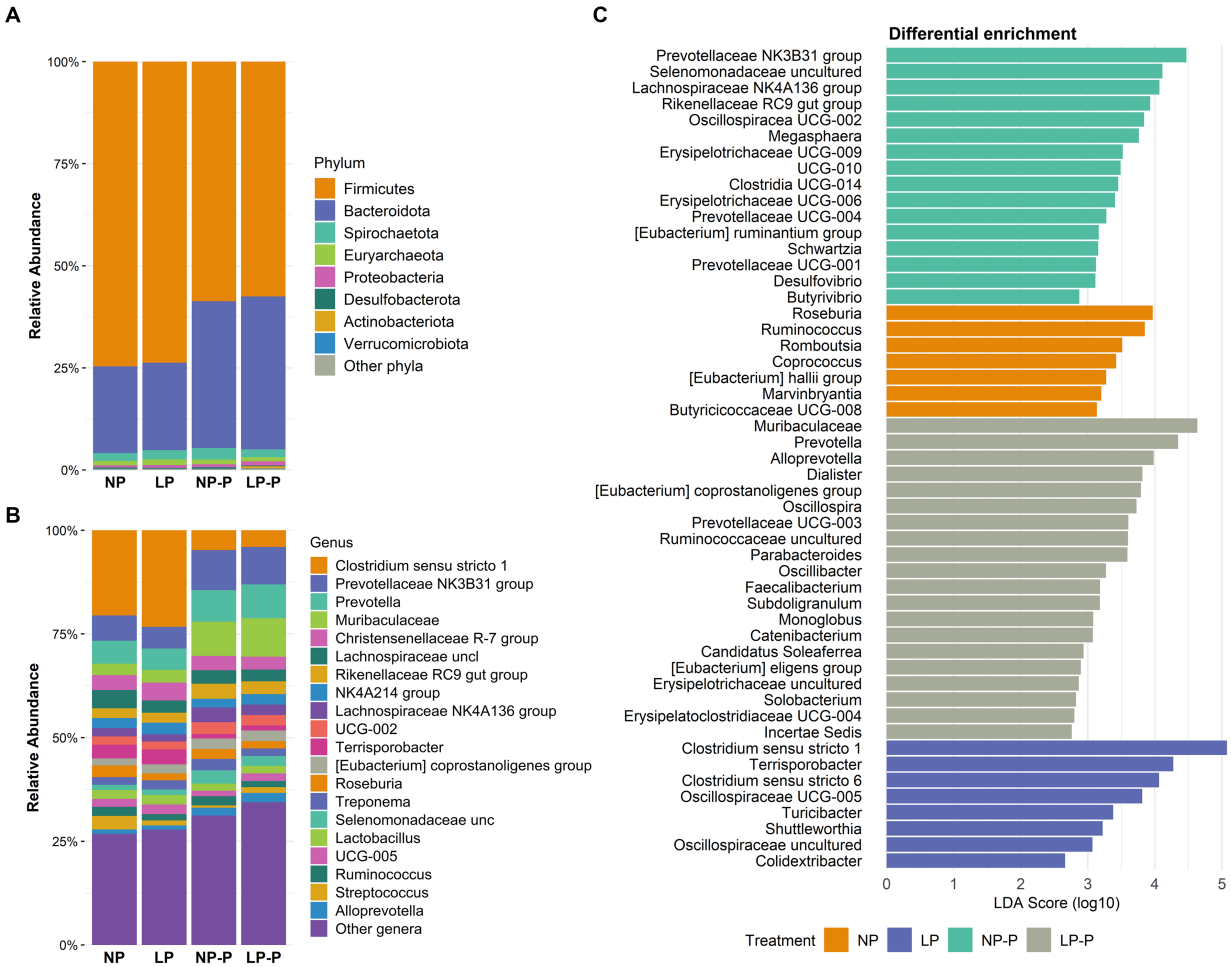
Changes in the gut microbiome composition. Relative abundance (%) at phylum **(A)** and genus **(B)** levels. Differential abundance analysis (LEfSe) showing taxonomical features identified among treatments **(C)**. NP, normal-level protein diet; LP, low-level protein diet; NP-P, normal-level protein diet + probiotic; LP-P, low protein diet + probiotic.

Moreover, we observed changes in the gut microbiome composition of the growing pigs at the phylum and genus levels. At the phylum level (Figure 2A; Supplementary Table S2), probiotic supplementation reduced the abundance of Firmicutes (*p* < 0.001) whereas increased that of Bacteroidota (*p* = 0.38), compared with that of the groups without probiotic supplementation. Desulfobacterota was enriched in both the NP and NP-P groups (0.28 and 0.33%, respectively; *p* < 0.001). Moreover, the abundance of several bacterial genera increased in the probiotic groups (Figure 2B; Supplementary Table S3). Irrespective of CP levels, *Prevotella* NK3B31 group (*p* < 0.001), *Prevotella* (*p* = 0.001), Muribaculaceae (*p* < 0.001), Rikenellaceae RC9 group (*p* < 0.001), *Eubacterium coprostanoligenes* group (*p* < 0.001), uncultured Selenomonadaceae (*p* < 0.001), and *Alloprevotella* (*p* < 0.001) were significantly enriched by probiotic supplementation. The abundance of Lachnospiraceae NK4A136 was enriched by probiotic supplementation (*p* < 0.001), but a higher increase was observed in case of normal-CP diet. Conversely, the abundance of *Clostridium sensu stricto* 1 and *Terrisporobacter* was reduced in the LP-P and NP-P groups (*p* < 0.001). On the other hand, both NP and NP-P enriched the abundance of *Roseburia* (*p* = 0.004) and *Ruminococcus* (*p* = 0.004); LP and LP-P had higher population of *Dialister* (*p* < 0.001) and *Megasphaera* (*p* = 0.003).

To examine the taxonomic markers in each treatment group, differential abundance analysis was performed using LEfSe (Figure 2C). NP diet differentially enriched *Roseburia, Ruminococcus, Romboutsia, Coprococcus, Eubacterium hallii, Marvinbryantia,* and Butyricicoccaceae UCG-008; the LP group exhibited differential abundance of *Clostridium sensu stricto* 1 and 6*, Terrisporobacter*, and uncultured Oscillospiraceae. Meanwhile, NP-P group was abundant in Prevotellaceae NK3B31 group, uncultured Selenomonadaceae, Lachnospiraceae NK4A136 group, *Eubacterium ruminantium*, and *Butyrivibrio*; and the LP-P group had differentially abundant Muribaculaceae, *Prevotella, Oscillospira, Alloprevotella*, and *Catenibacterium*.

### 3.4. Associations between the gut microbiome and fecal metabolites

**Figure 3:**
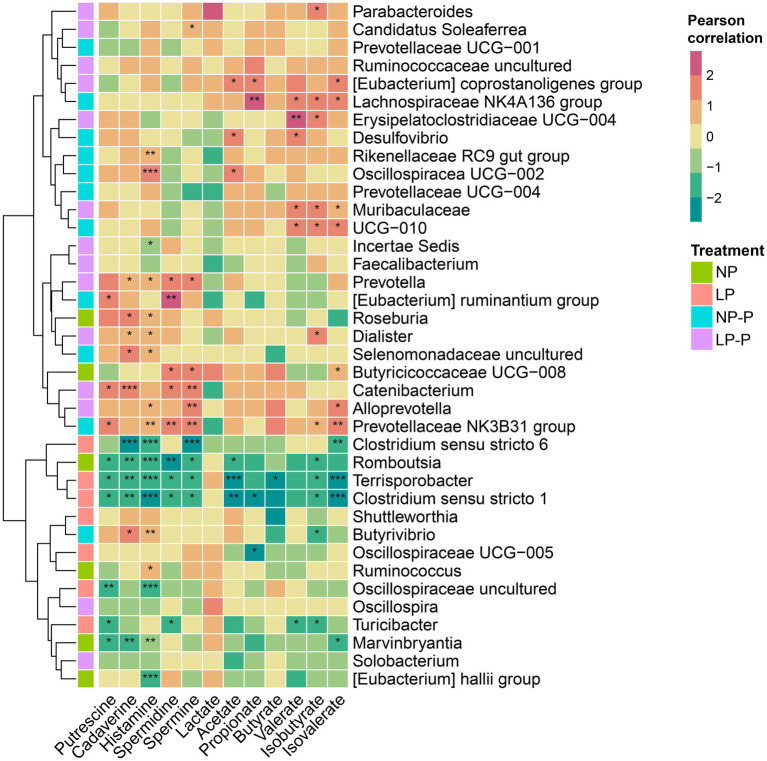
Gut microbiome composition correlates with fecal metabolites. Heatmaps based on Pearson correlation coefficients showing the relationship between gut microbiome and fecal metabolite levels (SCFAs, BCFAs, lactate, polyamines). *, **, and *** represent *p* < 0.05, *p* < 0.01, and *p* < 0.001, respectively. NP, normal-level protein diet; LP, low-level protein diet; NP-P, normal-level protein diet + probiotic; LP-P, low-level protein diet + probiotic.

Next, we examined the association between the gut microbiome and fecal metabolites. Pearson’s correlation coefficients were calculated and visualized using a heatmap (Figure 3). *Prevotella, Eubacterium ruminantium, Catenibacterium, Alloprevotella*, Prevotellaceae NK3B31 group, *Roseburia, Butyrivibrio, Dialister*, and uncultured Selenomonadaceae were positively correlated with fecal polyamine concentrations. Oscillospiraceae UCG-002, *Eubacterium coprostanoligenes*, Erysipelatoclostridiaceae UCG-004, *Desulfovibrio,* Lachnospiraceae NK4A136 group, Muribaculaceae, and UCG-010 have positive associations with SCFAs. BCFAs (isobutyrate and isovalerate) were positively associated with the abundances of *Parabacteroides*, *Alloprevotella,* Prevotellaceae NK3B31, Erysipelatoclostridiaceae UCG-004, Lachnospiraceae NK4A136 group, Muribaculaceae, UCG-010, and Butyricicoccaceae UCG-008. By contrast, *Romboutsia, Clostridium sensu stricto* 1 and 6, uncultured Oscillospiraceae, *Terrisporobacter, Marvinbryantia, Eubacterium hallii* group, and *Turicibacter* were negatively associated with fecal polyamines, SCFAs, and BCFAs. Moreover, it is noteworthy that the gut microbiota enriched by the addition of the multispecies probiotic (either NP-P or LP-P) were generally positively associated with fecal metabolites, and conversely, NP- or LP-abundant microbiota had an inverse relationship with fecal metabolites.

### 3.5. Prediction of the functional markers of the gut microbiome

**Figure 4:**
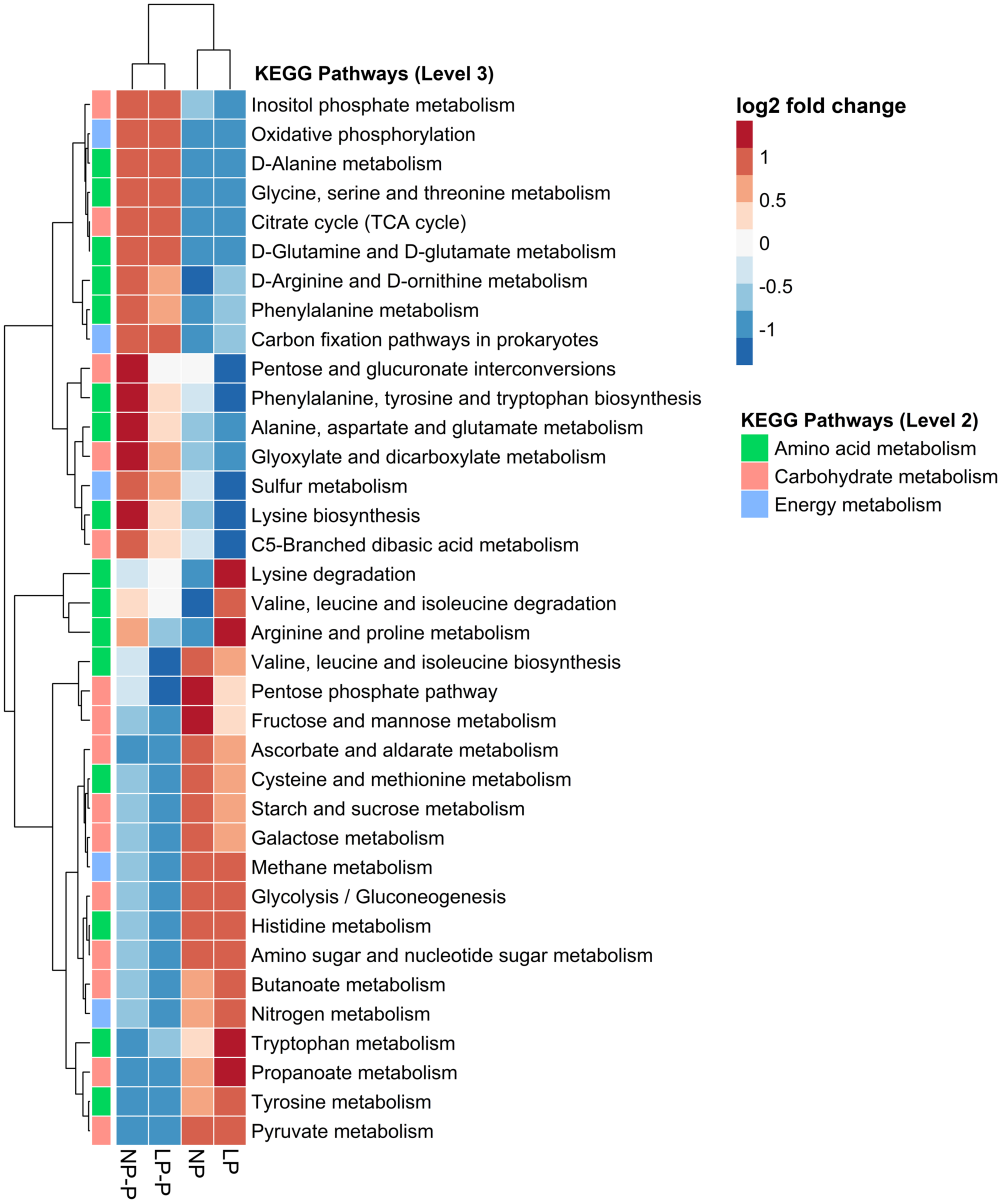
Changes in the functional capacity of the gut microbiome. Heatmap showing the relative abundances of metabolism-related KEGG pathways among different groups. NP, normal-level protein diet; LP, low-level protein diet; NP-P, normal-level protein diet + probiotic; LP-P, low-level protein diet + probiotic.

To assess the effects of multispecies probiotic supplementation in diet on the function of the gut microbiome of growing pigs fed different CP levels, metabolic pathways were predicted using PICRUSt2 and the KEGG database. KEGG pathways for metabolism (amino acid, carbohydrate, and energy) were selected and visualized in a heatmap (Figure 4). The addition of multispecies probiotics altered the predicted function of the gut microbiome with respect to metabolism. Several amino acid metabolism pathways showed greater abundance in the NP-P and LP-P groups, such as the metabolism of D-alanine, glycine, serine, and threonine, D-glutamine, and D-glutamate, D-arginine, and D-ornithine, phenylalanine, alanine, aspartate, and glutamate. Whereas metabolism of cysteine, methionine, histidine, tryptophan, and tyrosine was enriched in the NP and LP groups. Moreover, carbohydrate metabolism pathways were the most predicted in the NP and LP groups, such as the pentose phosphate pathway, fructose and mannose metabolism, starch and sucrose metabolism, galactose metabolism, amino sugar and nucleotide sugar metabolism, and propanoate metabolism. Finally, energy harvesting pathways, such as sulfur metabolism, were enriched in the NP-P and LP-P groups, whereas nitrogen metabolism was enriched in the NP and LP groups.

## 4. Discussion

In the present study, we observed that multispecies probiotic supplementation in diet significantly modulated the gut microbiome structure of growing pigs; however, similar effects were not observed by different CP levels in the diet. These results are in accordance with the results reported by Tang et al. (2019) who showed that probiotics have a more significant effect on the gut microbiome of pigs than dietary CP levels. A meta-analysis data revealed that CP level reduction by 2–3% may not be sufficient to influence the gut microbiota, especially in a short period of feeding (Luise et al., 2021). Yu et al. (2019) reported that reduction of CP from 17 to 14% did not alter the colonic microbiota of pigs. Moreover, the lack of difference between the microbiota of NP and LP might be influenced by the chosen sample in the current study (i.e., feces versus ileum), as pointed out by Pollock et al. (2019).

In addition to the composition of the gut microbiome, microbial-derived metabolites, such as SCFAs, BCFAs, and polyamines, also affect the intestinal health of the host. SCFAs, mainly butyrate, propionate, and acetate, are produced in the proximal colon as a product of fiber and, to some extent, protein degradation (Davila et al., 2013; Louis and Flint, 2017). Recent studies have demonstrated that these metabolites play a role in regulating homeostasis in the host gastrointestinal tract (GIT). SCFAs stimulate cell proliferation, suppress proinflammatory cytokines, and enhance the expression of tight junction proteins (Grilli et al., 2016; Zhong et al., 2019; Han et al., 2020; Xu et al., 2020). A reduction in dietary CP level could reduce the capacity of gut commensals to produce beneficial metabolites due to impaired fermentation capacity (Luise et al., 2021). Therefore, this could be an unwanted effect of the CP reduction strategies. In the present study, we observed a decrease in the levels of SCFAs, such as propionate and valerate, with decreasing CP level, which has been observed in previous studies (Chen et al., 2018; Yu et al., 2019). However, the addition of multispecies probiotic increased the levels of these SCFAs, especially in LP-P group, indicating that probiotics restored the capacity of the gut microbiome to produce SCFAs. In contrast, the addition of multispecies probiotics did not significantly alter the levels of fecal lactate, potentially since there were no changes in the dietary levels of carbohydrates. Like SCFAs, BCFAs, such as isobutyrate and isovalerate, are important bacterial metabolites. Although BCFAs are associated with protein fermentation (Rist et al., 2013; Rios-Covian et al., 2020), low CP level or probiotic supplementation did not exert any noticeable effect on BCFAs in the present study. Correlation analysis revealed that Oscillospiraceae UCG-002, *Eubacterium coprostanoligenes,* Lachnospiraceae NK4A136 group, Muribaculaceae, and Oscillospiraceae UCG-010 were positively correlated with SCFA and BCFA levels. In contrast, unclassified Prevotellaceae, Prevotellaceae NK3B31, and *Alloprevotella* were positively correlated with only BCFA levels. The bacterial family Lachnospiraceae and several members of the genus *Eubacterium* produce SCFAs (Biddle et al., 2013; Mukherjee et al., 2020). Moreover, Muribaculaceae and Oscillospiraceae are associated with high SCFA production (Smith et al., 2021; Yang et al., 2021; You et al., 2022). *Prevotella* and *Eubacterium* are markers for SCFA and BCFA production (Trefflich et al., 2021). Supplementation with probiotics has been shown to increase the capacity of the gut microbiome to produce SCFAs owing to the proliferation of metabolite-producing bacteria in the GIT (Sakata et al., 2003).

Polyamines are the products of amino acid fermentation by the gut microbiota (Matsumoto and Benno, 2007; Tofalo et al., 2019). Polyamine production by the microbiome is highly affected by the amount and source of dietary proteins (Rist et al., 2013; Wen et al., 2018). Moreover, as microbial metabolites, polyamines have important regulatory functions, such as the promotion of the small intestine development in piglets, suppression of inflammation (Liu et al., 2019), and regulatory functions in swine gestation (Wu et al., 2005). Polyamines exert beneficial effects on swine production, such as increasing growth and alleviating diarrhea symptoms (Van Wettere et al., 2016; Liu et al., 2019). However, excessive amounts of polyamines are linked with intestinal damage and occurrence of diarrhea in piglets (Ewtushik et al., 2000; Teti et al., 2002; Pieper et al., 2015). Polyamines may also contribute to the production of malodorous excretions in pigs (Jang and Jung, 2018). Some studies have reported that probiotics could affect the levels of polyamines in the GIT, although the results were inconsistent (Matsumoto et al., 2011; Ding et al., 2021). We observed small increase in polyamine levels in the LP-P group, indicating limited capacity of the multispecies probiotic to restore polyamine production by the gut microbiome. Nevertheless, correlation analysis showed several gut commensals were positively associated with fecal polyamine concentrations. *Prevotella* produces polyamines such as spermidine (Hanfrey et al., 2011; Nelson et al., 2015), and *Eubacterium* and *Ruminococcus,* both members of the phylum Firmicutes, could metabolize amino acids, such as ornithine, arginine, and lysine, to produce polyamines (Li et al., 2021). *Roseburia* and *Dialister* can also produce polyamines (Nelson et al., 2015; Tamanai-Shacoori et al., 2017). Our results strongly suggest that low CP diet could reduce beneficial gut microbiome metabolites, but the addition of probiotics could restore metabolite production by the gut commensals in low CP diet.

Moreover, we observed that reduction in dietary CP level also influenced the function of the gut microbiome, which is consistent with the results of previous studies (Liu et al., 2021; Tao et al., 2021). Pathways related to amino acid metabolism were enriched in the LP-P and NP-P groups compared with the NP or LP groups. This strongly suggests that probiotics can restore the ability of the gut microbiome to utilize undigested proteins in the colon, thereby enhancing the production of SCFAs and polyamines. This is consistent with the idea that probiotics enhance the protein digestion capacity of the host (Wang and Ji, 2018; Peng et al., 2019; Akhtar et al., 2022). Furthermore, the associations of several protein-utilizing commensals, such *Prevotella, Alloprevotella, Eubacterium*, and Murabaculaceae with protein-related metabolites, could account for the enrichment of amino acid metabolism pathways. Hence, we hypothesize that the enrichment of pathways for amino acid metabolism was favored rather than the carbohydrate pathways. The metabolism of amino acids such as glycine, threonine, glutamate, alanine, and aspartate, by colonic bacteria can produce SCFAs, whereas the metabolism of lysine, arginine, and ornithine produces polyamines (Zhao et al., 2018). Furthermore, supplementation with probiotics favored biosynthetic pathways, such as the TCA cycle; biosynthesis of phenylalanine, tyrosine, and tryptophan; biosynthesis of lysine; and pentose and glucuronate interconversion. According to Portune et al. (2016), the function of the microbiome can shift to the utilization of nitrogenous compounds for biosynthetic pathways when there is an abundance of other energy sources.

The fecal pH indicates the fermentation capacity of the colon. Lower colonic pH is beneficial for gut health as it can suppress the growth of potential pathogens, enhance protein digestion, and may indicate increased production of gut microbiome metabolites, such as SCFAs (Wen et al., 2018; Tang et al., 2019). We observed that supplementation with multispecies probiotic resulted in a significant decrease in fecal pH, which is in accordance with results of previous studies on the effect of probiotics on colonic pH (Pereira et al., 2022). A reduction in dietary CP level or probiotic supplementation improves the fecal score of pigs (Balasubramanian et al., 2018; Lu et al., 2018; Wen et al., 2018). Moreover, in the present study, the addition of multispecies probiotics to low CP diets significantly improved the fecal moisture content and fecal scores of growing pigs. This suggests that combinatory use of multispecies probiotics with CP reduction can ameliorate watery stools in growing pigs.

This study has limitations. First, the effect of reduction in dietary CP may have a more definitive effect on other segments of the GIT than in colon, which was not explored by the current study. In addition, the effects of low CP diet with multispecies probiotics on the actual protein digestion or absorption were not measured as this study focused mainly on the gut microbiome.

## 5. Conclusion

In the present study, we demonstrated that the combination of low CP diet and multispecies probiotic supplementation improved the fecal scores of the pigs. Moreover, pigs fed with multispecies probiotics had a distinct microbiome structure and composition. Pigs fed with low CP diet with multispecies probiotics had higher species richness and diversity. Multispecies probiotic supplementation in feed with low CP also altered the protein digestion and utilization activity of the gut microbiome, potentially contributing to higher fecal concentrations of SCFAs and marginally elevated polyamine levels. Population of gut microbiota such as Oscillospiraceae UCG-002, *Eubacterium coprostanoligenes*, Lachnospiraceae NK4A136 group, Muribaculaceae *Prevotella, Eubacterium ruminantium, Catenibacterium, Alloprevotella*, Prevotellaceae NK3B31 group, *Roseburia, Butyrivibrio*, and *Dialister* were associated with SCFAs and polyamine levels. To the best of our knowledge, this is the first study to describe the effects of multispecies probiotic supplementation and low CP diet on gut microbiome function. Therefore, supplementation with multispecies probiotics may complement the beneficial effects of low CP levels in pig feed. These findings may help formulate sustainable feeding strategies for swine production. The effects of this combined approach on the quality of animal production and environmental aspects must be further validated.

## Data availability statement

The datasets presented in this study can be found in online repositories. The names of the repository/repositories and accession number(s) can be found in the article/Supplementary material.

## Ethics statement

The animal study was reviewed and approved by Dankook University Animal Care Committee.

## Author contributions

D-KK and IK: conceptualization. RV: data curation, visualization, and writing – original draft. RV and SK: formal analysis and software. D-KK, IK, and JO: methodology. RV, SK, JO, I-CH, and JS: investigation. D-KK: writing – review and editing. All authors contributed to the article and approved the submitted version.

## Funding

This work was supported by the Korea Institute of Planning and Evaluation for Technology in Food, Agriculture, Forestry and Fisheries (IPET) through the Technology Commercialization Support Program, funded by the Ministry of Agriculture, Food and Rural Affairs (MAFRA) (Grant No: 122038-2).

## Conflict of interest

The authors declare that the research was conducted in the absence of any commercial or financial relationships that could be construed as a potential conflict of interest.

## Publisher’s note

All claims expressed in this article are solely those of the authors and do not necessarily represent those of their affiliated organizations, or those of the publisher, the editors and the reviewers. Any product that may be evaluated in this article, or claim that may be made by its manufacturer, is not guaranteed or endorsed by the publisher.
