# Supplementary material for: Multispecies probiotic supplementation in diet with reduced crude protein levels altered the composition and function of gut microbiome and restored microbiome-derived metabolites in growing pigs
Source: Front Microbiol. 2023 Jul 7;14:1192249. doi: 10.3389/fmicb.2023.1192249 (PMC10360209; doi:10.3389/fmicb.2023.1192249)
Supplement: Supplementary file 1 [file Data_Sheet_1.PDF]

Supplementary Material

Multispecies probiotic supplementation in diet with reduced crude protein levels altered the composition and function of gut microbiome and restored microbiome-derived metabolites in growing pigs

Robie Vasquez[1], Sang Hoon Kim[2], Ju Kyoung Oh[3], Ji Hoon Song[1], In-Chan Hwang[1], In Ho Kim[1], and Dae-Kyung Kang[1]*

*** Correspondence:**
Dae-Kyung Kang, Ph.D.
dkkang@dankook.ac.kr

Table S1. Alpha diversity measurements.

Item	Treatments[1,2] (%)				*p* value[3]		
	NP	LP	NP-P	LP-P	CP	Pro	CP × Pro
Chao1	783.2[a]	734.1[a]	790.8[a]	1557.0[b]	<0.001	<0.001	<0.001
Shannon	7.2[a]	7.1[a]	7.7[a]	8.3[b]	0.02	<0.001	0.003
Simpson	0.96[a]	0.95[a]	0.99[b]	0.99[b]	0.93	<0.001	0.21

[1]Values were reported as mean (n = 35 each treatment).
[2]Signifcant differences among treatments were determined using Kruskal-Wallis test. Mean values within a row without a common superscript differ significantly ($p < 0.05$).
[3]*p* values were calculated using multivariate ANOVA (MANOVA).
Abbreviations: NP, Normal-level protein diet; LP, Low-level protein diet; NP-P, Normal-level protein diet + probiotics; LP-P, Low-level protein diet + probiotics; CP, crude protein; Pro, probiotics.

Table S2. Relative abundance of fecal microbiota at phylum level.

Phyla	Treatments[1,2] (%)				SEM	*p* value[3]		
	NP	LP	NP-P	LP-P		CP	Pro	CP × Pro
Firmicutes	74.62[a]	73.75[a]	58.69[b]	57.52[b]	0.010	0.442	<0.001	0.914
Bacteroidota	21.23	21.34	35.93	37.42	0.002	0.734	0.38	0.294
Spirochaetota	1.92[a]	2.25[a]	2.83[b]	1.93[b]	0.010	0.552	<0.001	0.635
Proteobacteria	0.51	0.68	0.74	1.02	0.003	0.523	0.503	0.163
Desulfobacterota	0.28[ac]	0.13[b]	0.33[a]	0.19[bc]	0.001	<0.001	0.103	0.792
Actinobacteriota	0.02	0.05	0.05	0.49	0.001	0.594	0.869	0.426
Verrucomicrobiota	0.01[a]	0.08[a]	0.09[a]	0.10[b]	0.001	<0.001	<0.001	<0.001
Fibrobacterota	0.03	0.06	0.08	0.13	0.001	0.168	0.592	0.197
Campilobacterota	0.06[a]	0.06[ab]	0.05[ab]	0.09[b]	0.001	0.020	0.001	0.614
Cyanobacteria	0.03	0.04	0.03	0.04	0.001	0.687	0.014	0.988
WPS-2	0.05	0.05	0.001	0.01	0.001	0.189	0.710	0.957
Synergistota	0.01	0.01	0.011	0.02	0.001	0.596	0.108	0.082
Elusimicrobiota	0.003	0.01	0.008	0.005	0.001	0.592	1.000	0.083
Other phyla	0.008	0.003	0.022	0.005	0.010			

[1]Values were reported as mean (n = 35 each treatment).
[2]Signifcant differences among treatments were determined using Kruskal-Wallis test. Mean values within a row without a common superscript differ significantly ($p < 0.05$).
[3]*p* values were calculated using multivariate ANOVA (MANOVA).
Abbreviations: NP, Normal-level protein diet; LP, Low-level protein diet; NP-P, Normal-level protein diet + probiotics; LP-P, Low-level protein diet + probiotics; CP, crude protein; Pro, probiotics.

Table S3. Relative abundance of fecal microbiota at genus level. Cut-off set at 0.01%.

Genera	Treatments[1,2] (%)				SEM	*p* value[3]		
	NP	LP	NP-P	LP-P		CP	Pro	CP × Pro
Clostridium sensu stricto 1	20.53[a]	23.30[a]	4.79[b]	4.01[b]	0.013	0.625	<0.001	0.383
Prevotellaceae NK3B31 group	6.11[ab]	5.21[b]	9.67[a]	9.05[a]	0.005	0.344	<0.001	0.860
Prevotella	5.54[ab]	5.17[b]	7.59[a]	8.15[a]	0.004	0.900	0.001	0.539
Muribaculaceae	2.76[a]	3.07[a]	8.26[b]	9.29[b]	0.004	0.250	<0.001	0.536
Christensenellaceae R-7 group	3.59	4.32	3.46	3.09	0.004	0.804	0.365	0.460
Lachnospiraceae unclassified	4.42	2.94	3.22	2.83	0.002	0.009	0.066	0.125
Rikenellaceae RC9 gut group	2.33[ab]	2.37[b]	3.66[a]	3.09[ab]	0.002	0.454	0.004	0.378
NK4A214 group	2.43	2.82	2.12	2.51	0.002	0.087	0.168	0.988
Lachnospiraceae NK4A136 group	2.00	1.73	3.51	2.53	0.002	0.048	<0.001	0.250
UCG-002	2.03[ab]	1.88[b]	2.87[a]	2.54[ab]	0.002	0.328	0.002	0.716
Terrisporobacter	3.31[a]	3.63[a]	1.08[b]	1.20[b]	0.002	0.481	<0.001	0.745
Eubacterium coprostanoligenes group	1.66[a]	2.19[ab]	2.55[bc]	2.55[c]	0.001	0.112	<0.001	0.111
Roseburia	2.84[ab]	1.66[b]	2.37[a]	1.77[ab]	0.002	0.004	0.554	0.348
Treponema	1.86	2.21	2.73	1.83	0.003	0.525	0.572	0.156
Selenomonadaceae uncultured	1.21[a]	1.33[a]	3.22[b]	2.39[ab]	0.002	0.303	<0.001	0.175
Lactobacillus	2.14	2.30	1.71	1.83	0.002	0.704	0.206	0.951
UCG-005	1.94[ab]	2.33[b]	1.32[a]	1.83[ab]	0.001	0.009	0.001	0.729
Ruminococcus	2.18[a]	1.50[ab]	2.24[b]	1.47[b]	0.002	0.004	0.959	0.846
Streptococcus	3.27	1.19	0.54	1.38	0.004	0.330	0.047	0.023
Alloprevotella	1.06[a]	1.03[a]	1.88[b]	2.29[b]	0.001	0.282	<0.001	0.203
Clostridium sensu stricto 6	1.76[a]	2.27[a]	0.53[b]	0.81[b]	0.003	0.309	0.001	0.767
UCG-010	1.27[ab]	1.02[b]	1.46[a]	1.38[ab]	0.001	0.143	0.015	0.456
Lachnospiraceae AC2044 group	0.96	0.71	1.81	0.99	0.002	0.103	0.089	0.379
Methanobrevibacter	0.97	1.21	1.11	0.97	0.002	0.816	0.805	0.345
Oscillospira	0.60[a]	0.69[ab]	1.10[ab]	1.30[b]	0.001	0.358	0.001	0.756
Phascolarctobacterium	0.66	0.64	0.92	1.08	0.001	0.476	0.001	0.370
Prevotellaceae unclassified	0.69[a]	0.61[ab]	0.82[ab]	1.12[b]	0.001	0.535	0.070	0.294
Family XIII AD3011 group	0.68	0.82	0.83	0.86	0.001	0.163	0.136	0.375
Ruminococcaceae uncultured	0.52[a]	0.50[a]	0.85[b]	1.07[b]	0.001	0.159	<0.001	0.086

Selenomonadaceae unclassified	0.32^{a}	0.33^{a}	1.02^{b}	1.14^{b}	0.001	0.674	<0.001	0.725
Lachnospiraceae XPB1014 group	0.67	0.86	0.70	0.48	0.001	0.904	0.326	0.252
Clostridia UCG-014	0.63^{ab}	0.45^{b}	0.87^{a}	0.68^{a}	0.001	0.034	0.006	0.941
Dialister	0.14^{a}	0.37^{a}	0.99^{ab}	1.05^{b}	0.002	0.524	0.001	0.695
Parabacteroides	0.31^{a}	0.67^{bc}	0.38^{ab}	0.93^{c}	0.001	<0.001	0.109	0.361
Megasphaera	0.18^{a}	0.28^{a}	1.01^{ab}	0.81^{b}	0.002	0.821	0.003	0.510
Other genera	2.26	2.60	2.47	3.53	0.005			

[1]Values were reported as mean (n = 35 each treatment).
[2]Signifcant differences among treatments were determined using Kruskal-Wallis test. Mean values within a row without a common superscript differ significantly ($p < 0.05$).
[3]*p* values were calculated using multivariate ANOVA (MANOVA).
Abbreviations: NP, Normal-level protein diet; LP, Low-level protein diet; NP-P, Normal-level protein diet + probiotics; LP-P, Low-level protein diet + probiotics; CP, crude protein; Pro, probiotics.

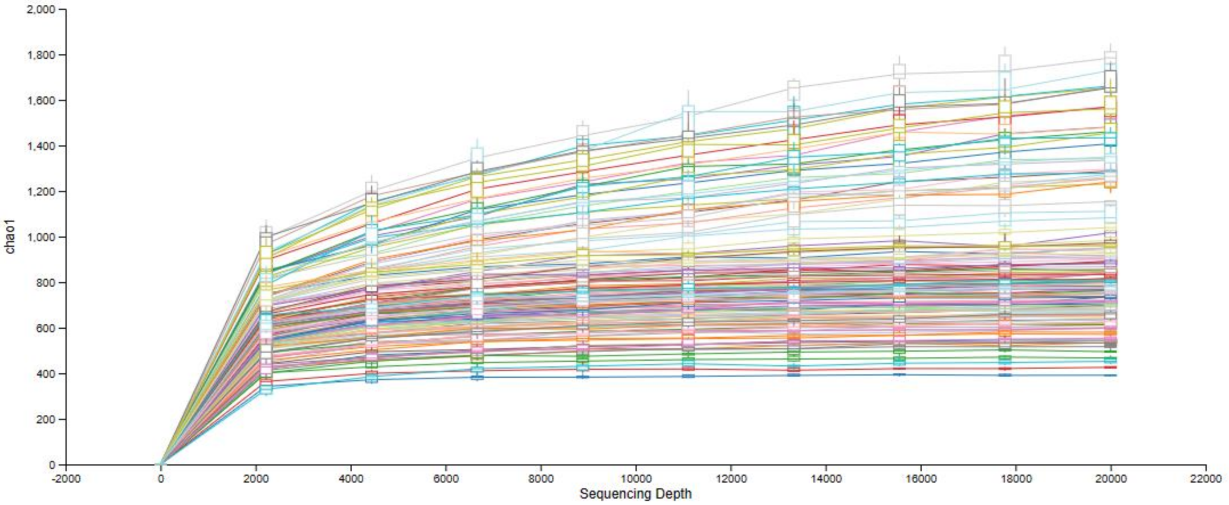

Figure S1. Alpha rarefaction curve based on Chao1 index.
